# Protocol for a Single-Arm Pilot Clinical Trial: Developing and Evaluating a Machine Learning Opioid Prediction & Risk-Stratification E-Platform (DEMONSTRATE)

**DOI:** 10.3390/jcm14238522

**Published:** 2025-12-01

**Authors:** Je-Won J. Hong, Debbie L. Wilson, Khoa Nguyen, Walid F. Gellad, Julie Diiulio, Laura Militello, Shunhua Yan, Christopher A. Harle, Danielle Nelson, Eric I. Rosenberg, Siegfried Schmidt, Chung-Chou Ho Chang, Gerald Cochran, Yonghui Wu, Stephanie A. S. Staras, Courtney Kuza, Wei-Hsuan Lo-Ciganic

**Affiliations:** 1 Department of Pharmacotherapy and Translational Research, College of Pharmacy, University of Florida, Gainesville, FL 32610, USA; 2 Department of Pharmaceutical Outcomes and Policy, College of Pharmacy, University of Florida, Gainesville, FL 32611, USA; 3 Division of General Internal Medicine, School of Medicine, University of Pittsburgh, Pittsburgh, PA 15213, USA; 4 Center for Pharmaceutical Policy and Prescribing, University of Pittsburgh, Pittsburgh, PA 15213, USA; 5 Center for Health Equity Research Promotion, Veterans Affairs Pittsburgh Healthcare System, Pittsburgh, PA 15240, USA; 6 Applied Decision Science, Cincinnati, OH 45212, USA; 7 Department of Health Policy and Management, Richard M. Fairbanks School of Public Health, Indiana University, Indianapolis, IN 46202, USA; 8 Center for Biomedical Informatics, Regenstrief Institute, Indianapolis, IN 46202, USA; 9 Department of Community Health and Family Medicine, College of Medicine, University of Florida, Gainesville, FL 32608, USA; 10 Division of General Internal Medicine, Department of Medicine, College of Medicine, University of Florida, Gainesville, FL 32608, USA; 11 Division of Epidemiology, Department of Internal Medicine, University of Utah, Salt Lake City, UT 84132, USA; 12 Health Outcomes and Biomedical Informatics, College of Medicine, University of Florida, Gainesville, FL 32611, USA; 13 Geriatric Research Education and Clinical Center, North Florida/South Georgia Veterans Health System, Gainesville, FL 32608, USA

**Keywords:** clinical decision support, machine learning, opioid overdose, primary care

## Abstract

**Background/Objectives**: The Developing and Evaluating a Machine Learning Opioid Prediction & Risk-Stratification E-Platform (DEMONSTRATE) trial aims to assess the usability, acceptability, feasibility, and effectiveness of implementing a machine learning (ML)-based clinical decision support (CDS) tool—the Overdose Prevention Alert—which predicts a patient’s risk of opioid overdose within three months. **Methods**: This single-arm study uses a pre–post implementation design with mixed-methods evaluation in 13 University of Florida Health, Gainesville, internal medicine and family medicine clinics. Eligible patients are aged ≥18 years, received an opioid prescription within the year prior to their upcoming primary care visit, are not receiving hospice care, do not have a malignant cancer diagnosis, and are identified by the ML algorithm as high risk for overdose. The Overdose Prevention Alert triggers when a primary care provider (PCP) signs an opioid order in electronic health records. We will evaluate effectiveness by comparing pre- and post-implementation outcomes using a composite patient-level measure defined by the presence of any of the following 6 favorable indicators: (1) evidence of naloxone access; (2) absence of opioid overdose diagnoses and naloxone administration; (3) absence of emergency department (ED) visits or hospitalizations due to opioid overdose or opioid use disorder (OUD); (4) absence of overlapping opioid and benzodiazepine use within a 7-day window; (5) absence of opioid use ≥50 morphine milligram equivalent daily average; (6) receipt of referrals to non-pharmacological pain management. Additional quantitative metrics will include alert penetration, usage patterns, and clinical actions taken. Usability and acceptability will be assessed using a 12-item questionnaire for PCPs and semi-structured interviews. **Expected Results**: The trial will provide insights into real-world ML-driven CDS implementation and inform future strategies to reduce opioid-related harm.

## 1. Introduction

In 2024, more than half of all drug overdoses in the United States (US) involved opioids [1]. Existing prevention strategies for opioid overdoses and opioid use disorder (OUD)—such as prescription drug monitoring programs (PDMPs) [2] and adherence to the Centers for Disease Control and Prevention’s opioid prescribing guidelines [3]—have primarily focused on prescribing practices. These include limiting high-dose opioids (e.g., <50 morphine milligram equivalents [MME]/day) and reducing the use of multiple prescribers. Although these efforts are important, prior studies have shown they often fail to accurately identify patients at the highest risk of overdose or OUD and have had mixed findings or limited impact on opioid overdose-related mortality [4,5,6]. For example, mandated PDMP reviews and pain clinic laws across 38 states achieved only a 12% reduction in overdose deaths between 2006 and 2013 [4], while Centers for Medicare & Medicaid Services’ (CMS’) simple rule-based criteria failed to capture 70% of beneficiaries who experienced an overdose [5]. A large community-based cluster-randomized trial aimed at reducing opioid overdose deaths—through multimodal communication campaigns promoting opioid overdose education, naloxone distribution, medications for OUD, and stigma-reduction messages—also found no significant difference in opioid overdose deaths between the intervention and control groups [6].

Early detection of patients at high risk for overdose is therefore essential. Device-based detection technologies, including contactless respiratory sensors and point-of-care monitors for opioid-induced respiratory depression, show promise for real-time physiologic assessment but still require additional safety testing and face cost and feasibility barriers to widespread use [7]. Current surveillance systems, such as PDMPs and national overdose surveillance networks [8], operate largely within separate data and resource domains. These systems rely on retrospective, rule-based criteria and often identify risk only after adverse events occur, underscoring the need for integrated, data-driven, and proactive approaches to identify high-risk patients before harm occurs. Motivated by these gaps, we developed a machine learning (ML)-based clinical decision support (CDS) platform designed to leverage routinely collected electronic health record (EHR) data to provide timely, individualized overdose risk predictions within primary care settings, enabling targeted intervention while preserving appropriate pain management.

This approach aligns with the recommendations from the 2017 President’s Commission on Combating Drug Addiction and the Opioid Crisis, which called for applying advanced data analytics to address the opioid epidemic [9]. We first developed and validated ML algorithms using diverse datasets, which outperformed conventional risk measures in predicting opioid overdose within a 3-month timeframe [5,10,11,12]. These models were trained on different populations including Medicare, Medicaid, and commercially insured populations using claims and integrated health and justice records. We then refined the ML algorithm using University of Florida (UF) Health’s Integrated Data Repository (IDR), a comprehensive clinical data warehouse linked to Epic^®^, the most widely used electronic health record (EHR) system in the US [13,14]. The UF Health training cohort included non-cancer and non-hospice adults (*n* = 130,136) aged ≥18 years who were prescribed ≥1 outpatient opioid between 2016 and 2020. We also examined algorithmic bias across key demographic subgroups to ensure model fairness and equitable performance prior to deployment [15].

Because primary care clinicians (PCPs) provide ongoing, longitudinal care and are key prescribers of opioids for chronic pain management, translating this ML model into a real-time decision-support tool required careful attention to workflow, usability, and provider engagement. To support integration into routine care, we embedded our ML algorithm in a CDS tool called the Overdose Prevention Alert. However, implementing CDS in clinical practice presents well-documented challenges, including alert fatigue, workflow disruptions, provider trust in ML outputs, and the potential for unintended consequences. To address these barriers and promote adoption, we used an iterative user-center-design approach to develop both the user interface (UI) and the backend implementation process for UF Health primary care clinics in Gainesville, Florida [16,17]. The “Developing and Evaluating a Machine Learning Opioid Prediction & Risk-Stratification E-Platform (DEMONSTRATE)” pilot trial aims to assess the usability, acceptability, and feasibility and impact of the Overdose Prevention Alert across 13 primary care clinics at UF Health Gainesville, Florida.

## 2. Material and Methods

### 2.1. Study Design

The DEMONSTRATE study is a single-arm pilot clinical, pre- and post-implementation study using a mixed-methods design to evaluate the outcomes of a CDS tool’s implementation. The study is guided by Proctor’s Implementation Outcomes, the CDS Five Rights, the Diffusion of Innovation Theory, the Consolidated Framework for Implementation Research (CFIR), and the Reach, Effectiveness, Adoption, Implementation, and Maintenance (RE-AIM) framework [18,19,20,21].

In alignment with the translational pathway for evaluating artificial intelligence (AI)–enabled CDS tools described by van der Vegt et al., who proposed the SALIENT (Systems Approach to Learning, Implementing, Evaluating, and Translating) framework [22], the DEMONSTRATE study corresponds to a Stage IV (“Field”) evaluation. This stage, consistent with the DECIDE-AI reporting guidelines [23] focuses on pilot, real-world implementation studies that assess usability, feasibility, and preliminary clinical impact prior to large-scale randomized controlled trials (Stage V, CONSORT-AI [24]). Accordingly, this pilot clinical trial was designed to obtain preliminary effect estimates and assess feasibility to inform a future, larger cluster-randomized clinical trial. Positioning this study within this structured translational continuum underscores the rationale for conducting a pragmatic, real-world pilot before broader deployment. A small prospective cluster-randomized controlled design was considered but deemed not feasible at this pilot phase, as leadership from the Departments of Internal Medicine and Community Health and Family Medicine recommended a unified rollout across all UF Health Gainesville primary care clinics to avoid confusion and ensure consistent implementation and clinician experience.

This trial was approved by the UF Institutional Review Board (IRB; UF IRB202002225, approval date: 13 October 2020), and registered on ClinicalTrials.gov (NCT06810076) [25]. This is a multi-institutional research project, where the UF IRB serves as the IRB of record for all participating sites, including the University of Pittsburgh (#STUDY24040038) and Applied Decision Science. This project is funded by the National Institutes of Health National Institute on Drug Abuse (R01DA050676). Protocol details follow the Standard Protocol Items: Recommendations for Interventional Trials (SPIRIT) checklist [26].

### 2.2. Setting, Participants and Recruitment

The implementation of the Overdose Prevention Alert began on 8 April 2025, in a convenience sample of 13 UF Health Gainesville primary care clinics (3 Internal Medicine and 10 Community Health and Family Medicine sites) without blinding. To promote engagement, we recruited PCP champions from UF Health Gainesville Internal Medicine and Community Health and Family Medicine clinics prior to CDS tool developing the Overdose Prevention Alert. Before implementation, each clinic designated a facilitator to support rollout and address operational challenges. We offered $500 administrative incentive to each clinic. Prior to launching the Overdose Prevention Alert, the research team conducted Zoom-based training sessions during Internal Medicine and Community Health and Family Medicine faculty and medical directors’ meetings. Instructional materials related to the Overdose Prevention Alert were distributed via email and in print to all PCPs at the participating clinics (Files S1–S5). For each clinic, we identified an administrative staff member for communicating with our team, answering questions from the PCPs, and distributing instructional materials.

Eligible PCPs include physicians, nurse practitioners, residents, and fellows practicing at participating clinics who are authorized to prescribe opioids. PCPs were informed through training sessions, emails, and printed materials that they may be contacted to complete a brief questionnaire (File S6) and participate in an interview (File S7). The study aims to recruit at least 13 PCPs (at least one from each participating clinic with an Overdose Prevention Alert interaction) to complete the questionnaire and interviews. PCPs who complete both interviews and surveys receive a $50 gift card. The UF IRB granted a full waiver of informed consent for accessing encrypted, limited patient EHR data and a waiver of documentation of informed consent to contact PCPs for interviews and questionnaires access.

### 2.3. Patient Eligibility Criteria for ML Overdose Risk Score Generation

Patients are eligible for ML Overdose risk score generation if they are aged ≥18 years, received at least one opioid prescription within the year preceding an upcoming clinic visit scheduled to occur within the next two weeks at one of the 13 participating clinics during the intervention period. Patients with malignant cancer diagnoses or those receiving hospice care are excluded from risk score generation or censored from alert interventions. Patients are flagged as being at elevated overdose risk if their ML-generated risk scores are ≥92.5th percentile of the scores in the training sample [5,12,16].

Given these criteria, patients must have some interaction with the UF Health system within the past year but are not required to have long-term continuity of care or chronic opioid therapy. This study represents the first deployment of an AI/ML-driven overdose prevention alert within UF Health. Before implementation, the UF Health system utilized a legacy opioid alert that suggested a naloxone order based on simple rule-based criteria (i.e., total opioid use ≥50 morphine milligram equivalence [MME] daily average, concurrent sedative or hypnotic use with any opioid prescription, any methadone order, or a naloxone order with a start date > 30 days prior), along with routine PDMP review. However, historical alert data were not retained to inform future overdose risk, and preliminary analyses suggested that clinicians found these legacy alerts overly frequent, inaccurate, and of limited clinical utility—factors that contributed to low adoption.

#### Overview of the ML Overdose Risk Prediction Model

The ML model underlying the Overdose Prevention Alert was developed and validated using 2016–2020 UF Health EHR data to identify patients at elevated risk for opioid overdose. The training cohort included non-cancer, non-hospice adults (*n* = 130,136) aged ≥18 years who received ≥1 outpatient opioid prescription during this period (mean age 49.2 ± 17.0 years). Most were female (60.7%) and White (63.7%), with an overdose event rate of 0.3% during this period. Chronic pain conditions were common, including osteoarthritis (56.8%), low back pain (42.4%), and migraine/headaches (25.3%). Mental-health and substance-use disorders were also prevalent, including anxiety (12.0%), mood disorders (10.6%), depression (8.4%), alcohol use disorder (1.6%), and opioid use disorder (0.9%). These characteristics highlight the real-world clinical complexity of the UF Health primary care population.

The final model incorporates 57 predictors across four domains: (1) patient demographics; (2) healthcare utilization (e.g., number of hospitalizations); (3) prescription characteristics (e.g., opioids, benzodiazepines, gabapentinoids, muscle relaxants, antidepressants); (4) comorbid diagnoses (e.g., mental-health conditions, liver disease). Risk elevation is determined by the combined influence of these predictors rather than any single factor. Model performance is evaluated semiannually and updated as needed. Fairness assessments conducted prior to deployment (15), observing no significant race or sex disparities. The model demonstrated strong discrimination (C-statistic > 0.80) and calibration in prior evaluations. In practice, approximately 1 in 50 patients with a prior opioid prescription are expected to trigger the alert, and among those identified about 1 in 333 may experience an overdose within three months (versus 1 in 2600 at baseline). To promote transparency and support clinician understanding of the AI/ML model, these details are also summarized in a Frequently Asked Questions resource (File S2).

### 2.4. ML-Driven Overdose Prevention Alert Intervention

#### 2.4.1. Alert User Interface (UI)

The Overdose Prevention Alert is an interruptive CDS tool embedded within the UF Health EHR that surfaces when a PCP signs an opioid order for patients identified as elevated risk by the ML model (see Section 2.3). The clinician-facing UI and backend integration were co-developed using a user-centered design and human factors approach in collaboration with leadership, IT specialists, and PCPs to define functional requirements and mitigate unintended bias. Additional details of the alert’s design and implementation process have been published previously [16,17].

**Figure 1:**
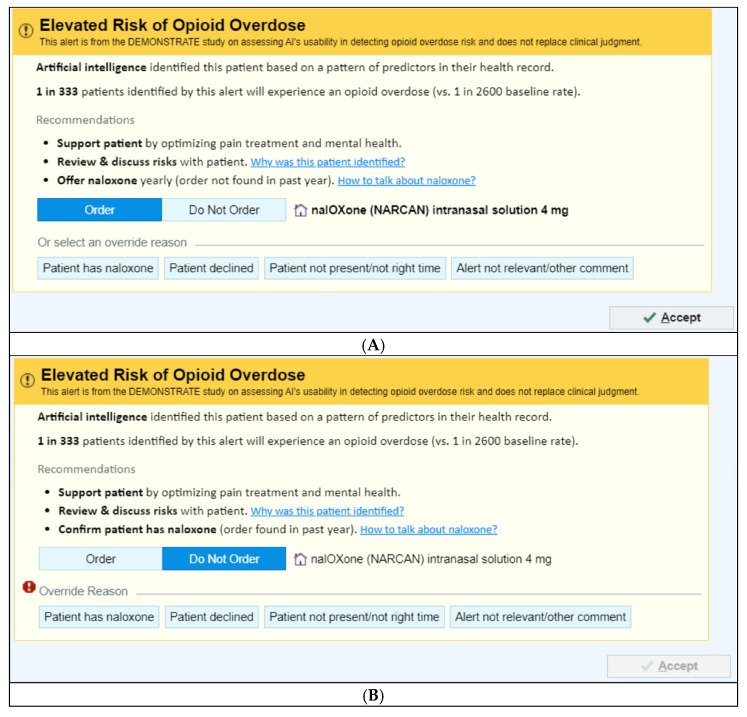
Image of the Overdose Prevention Alert User Interfaces. (**A**) Offer Naloxone © 2025 Epic Systems Corporation. (**B**) Confirm Naloxone © 2025 Epic Systems Corporation.

There are two UI versions depending on whether a naloxone order has been documented in the patient’s EHR in the past year:

(1) No naloxone order in the past year—“Order Naloxone Overdose Prevention Alert” (Figure 1A): Recommends ordering naloxone and provides three mitigation strategies:
  - “Support patient by optimizing pain treatment and mental health”;
  - “Review & discuss risks with patient”;
  - “Offer naloxone yearly (order not found in past year).”
(2) Naloxone order in the past year—“Confirm Patient Has Naloxone Overdose Prevention Alert” (Figure 1B): Defaults to “Do Not Order” and prompts the PCP to verify that the patient already possesses naloxone.

Both alert versions link to a Frequently Asked Questions resource (File S2) containing additional details on the ML risk prediction including input variables, predictors included, misclassification rates, fairness assessments, supporting evidence and limitations. A brief summary of the model is also provided in Section Overview of the ML Overdose Risk Prediction Model.

Each alert also includes a clinician communication script (File S3) to support concise discussions about opioid overdose risk and naloxone.

#### 2.4.2. Alert Exposure and Workflow

**Figure 2:**
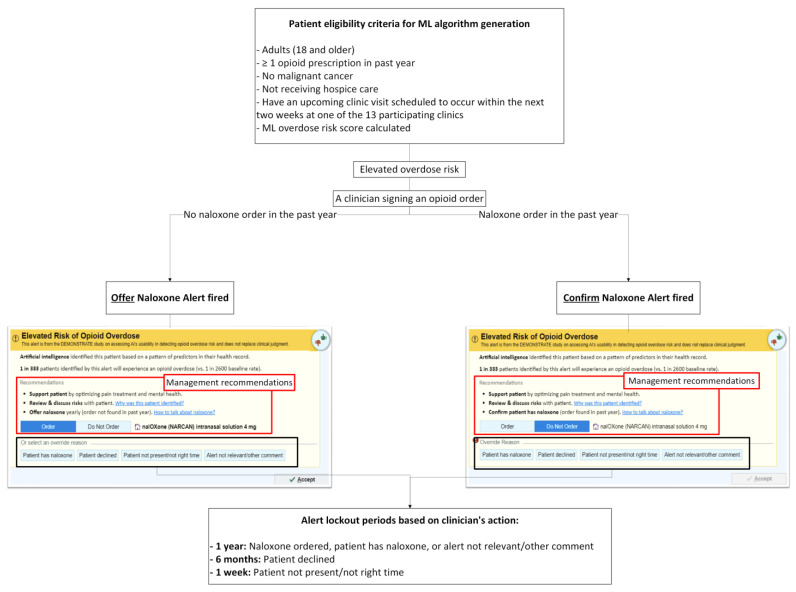
Workflow for Alert Triggering, Clinician Actions, and Alert Lockout Periods.

Figure 2 depicts the workflow logic of the Overdose Prevention Alert. The intervention is implemented at the clinic level. When a PCP signs an opioid prescription for a patient identified as high risk by the ML model, the system evaluates whether the patient has a documented naloxone order/fill within the past year and fires one of two alert versions accordingly. Because UF Health requires evidence of effectiveness before retiring the existing legacy naloxone alert, the legacy alert remains active during the study period. As a result, the legacy alert will continue to appear for patients who are not identified as high risk by the ML model but who meet the legacy alert’s simple rule-based criteria (i.e., total opioid use ≥50 MME/day, concurrent sedative or hypnotic use with any opioid prescription, any methadone order, or a naloxone order with a start date more than 30 days prior).

(1) **No naloxone order/fill record in the past year:** When a patient has not had a naloxone order/fill in the past year, “Order Naloxone Overdose Prevention Alert” (Figure 1A) is triggered at the time the PCP signs an opioid order. This version recommends naloxone prescribing and presents three management strategies:
  - “Support patient by optimizing pain treatment and mental health”;
  - “Review & discuss risks with patient”;
  - “Offer naloxone yearly (order not found in past year).”
  The default selection is “Order Naloxone.” PCPs may override by selecting “Do Not Order” or choosing a pre-labeled override reason, including:
  - “Patient has naloxone”,
  - “Patient declined”,
  - “Patient not present/not the right time”,
  - “Alert not relevant/other comment” (free text required).
(2) **Naloxone order/fill documented in the past year:** When a patient had a naloxone order/fill in the past year, “Confirm Patient Has Naloxone Overdose Prevention Alert” (Figure 1B) appears when the PCP signs an opioid order. This version defaults to “Do Not Order” and prompts the PCP to verify that the patient possesses naloxone. If the PCP elects to prescribe naloxone, they must actively select “Order.” PCPs may choose a pre-labeled override reason, including: “Patient has naloxone”, “Patient declined”, “Patient not present/not the right time,” and “Alert not relevant/other comment”.

In all cases, clicking “Accept” finalizes the action.

Alert lockout periods: To reduce alert fatigue, the alert is suppressed for different durations depending on the PCP’s action (illustrated in Figure 2):

- 1 year—Naloxone ordered, or a PCP chooses an override reason “Patient has naloxone” or “Alert not relevant/other comment.” Free-text entry is enabled for “Alert not relevant/other comment”.
- 6 months— A PCP chooses an override reason “Patient declined.”
- 1 week— A PCP chooses an override reason “Patient not present/not the right time.”

### 2.5. Outcomes

The study assesses usability, acceptability, and feasibility of the CDS tool through evaluating patient- and PCP-level outcomes up to 12 months post-implementation using mixed method evaluations. In weekly meetings, our team developed a structured Excel (Microsoft, Redmond, WA, USA, 2021) workbook of spreadsheets to systematically define potential outcomes. Although validated measures such as the System Usability Scale and Perceived System Usefulness questionnaire exist for measuring end user experiences, we determined they did not align well with our study goals and might not be interpreted consistently by end users. Thus, we drew upon multiple implementation and usability frameworks—including Proctor’s Implementation Outcomes [18], the CDS Five Rights [20], CFIR [21], RE-AIM [19], Computer System Usability Questionnaire [27], and a work-centered approach [28]—to guide the identification and organization of the study’s outcome measures. For each proposed outcome, we specified its operationalization, including denominator, numerator, timeframe (e.g., 3, 6 and 12 months post-implementation), reporting format (e.g., frequency, average, median, range), data sources (e.g., EHR query, PCP interview), and feasibility of measurement. After multiple iterations, our team agreed on the final set of outcome measures.

Primary outcomes are detailed below. Additional quantitative metrics include alert penetration, usage patterns, and clinical actions taken. Qualitative analyses will also be conducted on free-text override comments submitted by PCPs.

#### 2.5.1. Primary Patient-Level Outcomes

Using EHR data and EPIC^®^ query, the primary patient-level outcome is a 6-month composite measure outcome post-implementation defined by the presence of any of six favorable indicators:

(1) Evidence of naloxone access (order, fill, or documentation of possession [e.g., PCP selection of button ‘patient has naloxone’]),
(2) Absence of opioid overdose diagnoses and naloxone administration,
(3) Absence of ED visits or hospitalizations due to opioid overdose or OUD,
(4) Absence of overlapping opioid and benzodiazepine use within a 7-day window,
(5) Absence of opioid use ≥50 MME daily average,
(6) Receipt of referrals to non-pharmacological pain management (e.g., physical therapy, chiropractic care).

Secondary analyses will also examine each component of the composite outcome separately and evaluate outcomes at additional time points (3 and 12 months post-implementation).

#### 2.5.2. Primary PCP-Level Outcomes

We will assess PCP-reported attitudes and experiences with the Overdose Prevention Alert using a 15-item questionnaire (File S6) administered via REDCap electronic data capture tools [29,30] hosted at UF, followed by a brief semi-structured interview (File S7). The 15-item questionnaire and interview guide were developed, with them both tailored to the needs of this study and adapted from prior work [20,27,28].

The questionnaire includes one binary item (yes/no), two open ended questions, and 12 Likert-scale items (4-point scale: 1 = Strongly Disagree to 4 = Strongly Agree) evaluating the acceptability, appropriateness, and feasibility of the Overdose Prevention Alert. These items assess perceptions including:

(1) the Overdose Prevention Alert’s information was clear,
(2) the alert was easy to use,
(3) the alert helps identify patients at elevated overdose risk,
(4) the alert helps understand patient’s overdose risk,
(5) the alert provides risk management recommendations,
(6) the alert identifies the right patients with elevated overdose risk,
(7) the alert notifies the correct healthcare team member (i.e., PCPs),
(8) a pop-up alert is an appropriate notification approach,
(9) signing an opioid order is the right time for the alert,
(10) alert frequency is appropriate,
(11) I prefer the Overdose Prevention Alert over the legacy naloxone alert (see picture),
(12) I want the Overdose Prevention Alert to continue to operate in my EHR.

The semi-structured interview (File S7) includes six questions focused on the relevance, response to, impact, and perceived usefulness of the Overdose Prevention Alert.

#### 2.5.3. Secondary Alert Use-Related Outcomes

To understand how PCPs interacted with the Overdose Prevention Alert and how the CDS tool functioned within real-world workflows, we will assess a set of alert use–related outcomes derived from EHR data, EPIC Workbench Reports, and chart review (as needed). These outcomes reflect key implementation constructs—penetration, adoption, and appropriateness—and correspond directly to the operational definitions outlined in File S8.

Alert use–related outcomes that measure different aspects of implementation (overall and by alert type) include:

- Penetration: Penetration outcomes measure the extent to which the alert reached its intended users and patients. These include the total number of alert appearances (overall and by alert version: “Order Naloxone” vs. “Confirm Naloxone”), the percentage of ML-flagged elevated-risk patients who received ≥1 alert, the average number of alerts per alerted patient, and the percentage of PCPs at participating clinics who encountered at least one alert.
- Adoption: Adoption outcomes evaluate the extent to which clinicians accepted or acted upon the alert recommendations. Measures include the percentage of alerts resulting in an accepted naloxone order, patient-level naloxone acceptance (≥1 accepted naloxone order per alerted patient), and the percentage of alerts for which a naloxone order was initially selected but ultimately unsigned (e.g., deleted after clicking “Accept”).
- Appropriateness: Appropriateness outcomes examine whether clinicians perceived the alert as relevant and useful in practice. Measures include the proportion of alerts associated with specific override reasons (“Patient has naloxone,” “Patient declined,” “Patient not present/not the right time,” and “Alert not relevant/other”), patterns of override reasons across clinics and PCPs, and the percentage of alerts accompanied by free-text override comments (with qualitative analysis).

Operational definitions, numerators, denominators, timeframes, and data sources for all alert use–related outcomes are provided in File S8. Similar analyses will be conducted for the existing legacy naloxone alert for comparative purposes.

### 2.6. Sample Size Estimation

Based on preliminary data, each participating clinic prescribes opioids to an estimated 30 to 150 patients per month, depending on clinic size. Over a 6-month period, across 13 clinics, assuming an even distribution of high-risk patients across PCPs, we expect each PCP will have at least 1–2 opportunities to use the Overdose Prevention Alert. We anticipate a sample size of 1000 participants including PCPs and patients (identified as high-risk patients by the ML algorithm, including those for whom the Overdose Prevention Alert does and does not fire).

### 2.7. Statistical and Qualitative Analyses

#### 2.7.1. Patient-Level Outcomes

Descriptive statistics (e.g., mean ± standard deviation [SD]) will summarize the patient and PCP characteristics and outcomes including 2-sample *t*-tests (or non-parametric Wilcoxon rank-sum test) for comparing continuous variables and chi-squared tests (or equivalent non-parametric methods) for comparing categorical variables.

Because both the ML-based Overdose Prevention Alert and the legacy naloxone alert remain active during the intervention period, we will classify patients into three mutually exclusive exposure groups based on their alert history. The ML Alert Group will include ML-identified elevated-risk patients who receive at least one ML-based Overdose Prevention Alert during follow-up. The Legacy Alert Only Group will include elevated-risk patients who never receive an ML alert but do receive at least one legacy naloxone alert based on its rule-based criteria (≥50 MME/day, concurrent sedative/hypnotic use, any methadone order, or a naloxone order with a start date > 30 days prior). The No-Alert Group will include elevated-risk patients who do not receive either alert. Because the ML-based Overdose Prevention Alert represents the primary intervention, we will classify patients who receive both alerts into the ML Alert Group. To further examine real-world alert pathways, we will conduct an exploratory sensitivity analysis that reassigns patients by the sequence in which alerts appear (e.g., legacy→ML vs. ML→legacy).

We will conduct three main analyses. First, we will conduct an interrupted time series analysis to assess aggregated biweekly or monthly patient-level outcomes before and after ML-based alert implementation, while recognizing that the legacy alert continues to operate concurrently. Second, we will use multi-level Cox proportional hazard models [31] to compare the time to occurrence of the composite outcome across the three exposure groups. To address competing risks such as death, malignant cancer, or admission to hospice care, we will estimate cumulative incidence functions for the composite endpoint. Third, we will use multi-level mixed models to assess changes in patient outcomes 6 months before and after implementation across three comparison groups, adjusting for clustering at clinic and PCP levels. These models will also allow us to estimate the intraclass correlation coefficient, which will inform sample size calculations for future large-scale cluster-randomized trials. Statistical significance will be evaluated using two-tailed tests with an alpha level of 0.05.

We will also assess secondary outcomes at 3 and 12 months post-implementation.

#### 2.7.2. PCP-Level Outcomes

We will use descriptive statistics to summarize responses to the 15-item questionnaire, including mean scores with standard deviations for all 4-point Likert-scale items. Because PCPs may encounter both the ML-based and legacy alerts during the study period, we will interpret questionnaire responses in the context of dual alert exposure rather than stratifying results by alert type. We will analyze qualitative data from semi-structured interviews and free-text override comments using descriptive content analysis. Human-factors experts on the research team will independently review and code transcripts, iteratively refine emerging themes, and finalize a thematic structure supported by representative quotations.

#### 2.7.3. Alert Use-Related Outcomes

We will assess alert use–related outcomes to understand how clinicians interact with the alerts and how the CDS tools function in routine practice. We will use descriptive statistics to summarize alert penetration, adoption, and appropriateness. Consistent with File S8, we will analyze alert use-related outcomes at the alert level and report them separately for the ML-based Overdose Prevention Alert and the legacy alert. This approach will allow us to compare alert firing patterns, override behaviors, naloxone-ordering actions, and clinicians’ perceptions of alert relevance.

### 2.8. Confidentiality and Safety Monitoring

Data are used solely for research and maintained confidentially. The investigative team review participant accrual and adverse event data weekly. The primary risk associated with this study is a potential breach of confidentiality; however, safeguards have been implemented to minimize this risk. Another concern is the potential for unintended bias in PCP decision-making due to interaction with the Overdose Prevention Alert. To mitigate this, human factors principles were applied during development, and PCP training emphasized that the Overdose Prevention Alert is designed to support—not replace—clinical judgment. The alert highlights elevated overdose risk but does not mandate specific actions.

## 3. Discussion

This single-arm pilot clinical trial will generate early, exploratory evidence on the usability, acceptability, feasibility, and preliminary effects of implementing the ML-driven Overdose Prevention Alert in primary care. The findings will help determine whether the alert can be integrated into routine workflows and whether further evaluation in a fully powered cluster-randomized pragmatic trial is warranted. By operating within real-world clinical practice, the study offers practical insights into implementation processes and PCPs’ engagement with an AI-enabled CDS tool.

This study aligns with the SALIENT framework’s Stage IV (“Field”) evaluation [22] and the DECIDE-AI guidelines [23], both of which emphasize feasibility, workflow integration, and early-stage assessment of AI-enabled CDS tools prior to randomized trials. The study’s design incorporates recommended safeguards for responsible deployment—such as transparency resources, override options, and clinician-facing explanations—to support human oversight and real-world performance monitoring. These design elements reflect emerging best practices for responsible implementation of AI in clinical settings [32].

A major contribution of this work is the application of implementation science frameworks such as RE-AIM [19] and CFIR [21] to examine the real-world integration of an ML-driven CDS tool across 13 primary care clinics. Insights from this evaluation may clarify workflow considerations, user preferences, and operational barriers that influence CDS adoption. During development, human factors methods and stakeholder input were used to minimize unintended consequences, and emphasize that the Overdose Prevention Alert is designed to support—not replace—clinical judgment [16,17]. As AI-driven CDS tools become more prevalent, ensuring they do not exacerbate disparities in care access or outcomes remains essential, particularly given potential gaps in predictive accuracy among smaller or under-represented patient subgroups.

A key limitation of this study arises from its single-arm pre–post design and the real-world constraints of implementation at UF Health. Primary care leadership requested deployment of the Overdose Prevention Alert across all primary care clinics in Gainesville to avoid workflow inconsistencies, making it infeasible to include a concurrent control group. Consequently, observed changes in outcomes cannot be definitively attributed to the intervention, and findings may be influenced by secular trends such as evolving prescribing practices, institutional policy changes, or external events occurring during the study period. Additionally, UF Health elected to maintain its legacy rule-based naloxone alert throughout the pilot, resulting in clinicians potentially encountering both alerts during routine care. This introduces analytic complexity, which we address by classifying elevated-risk patients into mutually exclusive exposure groups—ML Alert, Legacy Alert Only, and No Alert—based on actual alert history. To further describe real-world alert pathways, we also conduct an exploratory sensitivity analysis examining alert sequences (e.g., legacy→ML vs. ML→legacy). Finally, because the ML risk prediction algorithm was developed using UF Health data exclusively, generalizability may be limited, although fairness assessments did not reveal major race or gender disparities.

Pragmatic implementation also presents operational challenges that may affect generalizability. Variations in EHR capabilities, staffing models, and prescribing practices across sites may affect how the alert is used and perceived. Additionally, AI-driven CDS tools such as the Overdose Prevention Alert must navigate issues including alert fatigue, workflow integration, and provider responsiveness. The pilot offers an opportunity to identify and address these barriers, generating insights that can guide future CDS implementation strategies. The study may also be underpowered to detect differences in individual patient-level outcomes due to the pilot design and the decline in opioid prescribing since project initiation [33]. Power estimates were based on 2019–2020 data and may not fully reflect current prescribing patterns, the proportion of elevated-risk patients flagged by the ML algorithm (which balances false positive rates), or clustering effects. Nevertheless, the pilot study will provide essential effect-size estimates to inform sample size planning for future large-scale cluster randomized trials [34,35] if pilot findings indicate that the intervention is feasible and acceptable.

Any major protocol changes will be reviewed by the investigator team, approved by the IRB, and communicated to relevant stakeholders. Study updates and results will be made publicly available on ClinicalTrials.gov. Following the study completion, we will disseminate findings to health systems, clinicians, policymakers, research participants, and the public. Aggregated-level data from the final dataset will be shared after publication of the final results.

Findings from this pilot study will directly inform the design of a future cluster-randomized or pragmatic trial. The pilot will generate preliminary effect-size estimates for both the composite and individual patient-level outcomes, provide empirical intraclass correlation coefficients to quantify clustering at the clinic and PCP levels, and document alert penetration and adoption patterns that determine expected event rates. These parameters are essential for calculating sample size, specifying the number of clusters, and determining the follow-up duration required for a fully powered evaluation. In addition, the workflow and usability findings—such as alert frequency, override behaviors, and provider engagement—will guide refinements to the CDS tool to ensure that the future trial evaluates a stable and operationally feasible version of the intervention. Together, these pilot data will provide the foundational methodological inputs needed to plan a rigorous large-scale trial.

## Data Availability

The datasets generated or analyzed in this study are not publicly accessible per the University of Florida Health data use agreement.

## Supplementary Materials

The following supporting information can be downloaded at: https://www.mdpi.com/article/10.3390/jcm14238522/s1, File S1: DEMONSTRATE Epic Tip Sheet; File S2: DEMONSTRATE FAQ Artificial Intelligence Alert Opioid Overdose Risk; File S3: DEMONSTRATE 30 Second Patient Counseling Scripts; File S4: DEMONSTRATE Opioid Overdose Risk Pocket Card; File S5: DEMONSTRATE Centers for Disease Control and Prevention Guidelines on Opioid Prescribing Infographic; File S6: DEMONSTRATE Post-Implementation Questionnaire; File S7: DEMONSTRATE Post-Implementation Interview Guide. File S8: Alert Use-Related Outcome Measures.

## Abbreviations

The following abbreviations are used in this manuscript:

AI	Artificial intelligence
CFIR	Consolidated Framework for Implementation Research
DEMONSTRATE	Developing and Evaluating a Machine Learning Opioid Prediction & Risk-Stratification E-Platform
ED	Emergency department
EHR	Electronic health record
IDR	Integrated Data Repository
IRB	Institutional review board
MME	Morphine milligram equivalents
ML	Machine learning
OUD	Opioid use disorder
PCP	Primary care provider
PDMP	Prescription drug monitoring program
RE-AIM	Reach, Effectiveness, Adoption, Implementation, and Maintenance
SaMD	Software as a Medical Device
SPIRIT	Standard Protocol Items: Recommendations for Interventional Trials
UI	User interface
UF	University of Florida
US	United States
