# Supplementary material for: Protocol for a Single-Arm Pilot Clinical Trial: Developing and Evaluating a Machine Learning Opioid Prediction & Risk-Stratification E-Platform (DEMONSTRATE)
Source: J Clin Med. 2025 Dec 1;14(23):8522. doi: 10.3390/jcm14238522 (PMC12693449; doi:10.3390/jcm14238522)
Supplement: Supplementary file 1 [file jcm-14-08522-s001.zip › Supplementary File S1_DEMONSTRATE Epic Tip Sheet 20250904.pdf]

Epic Version Aug 2024

ELEVATED RISK OF OPIOID OVERDOSE ALERT

This alert appears when you sign an order for an opioid medication for a patient who has been identified by an artificial intelligence (AI) algorithm as having an elevated risk of opioid overdose, based on a pattern of predictors in their health record. The alert recommends you to (1) support the patient by optimizing their pain treatment and mental health (if indicated), (2) review and discuss opioid overdose risks with the patient, and (3) offer naloxone (Narcan) to the patient yearly or confirm that the patient still has access to naloxone (if already prescribed).

Appearance Criteria

There are two versions of the alert: (A) Offer Naloxone, and (B) Confirm Naloxone.

Alert A: Offer Naloxone

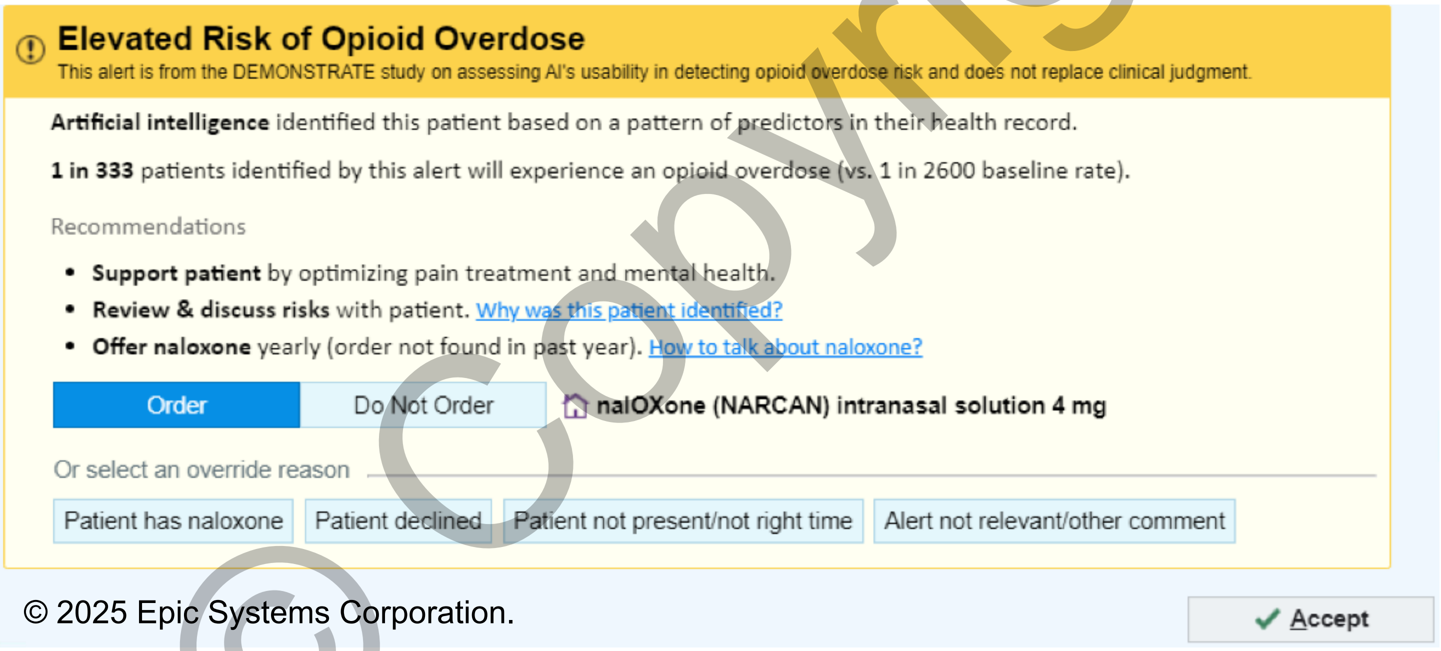

The 'Offer Naloxone' alert will appear when all the following trigger criteria have been met:

- ✓ The algorithm determines patient has elevated risk of opioid overdose.
- ✓ An order for an opioid medication has been entered for the patient.
- ✓ Naloxone is not part of the current order.
- ✓ Naloxone has not been ordered in the past year per Epic records.
- ✓ The provider signs the order (alert will appear on this action).

This alert is defaulted to 'Order' naloxone and can be accepted immediately by selecting the "Accept" button.

© Copyright

Alert B: Confirm Naloxone

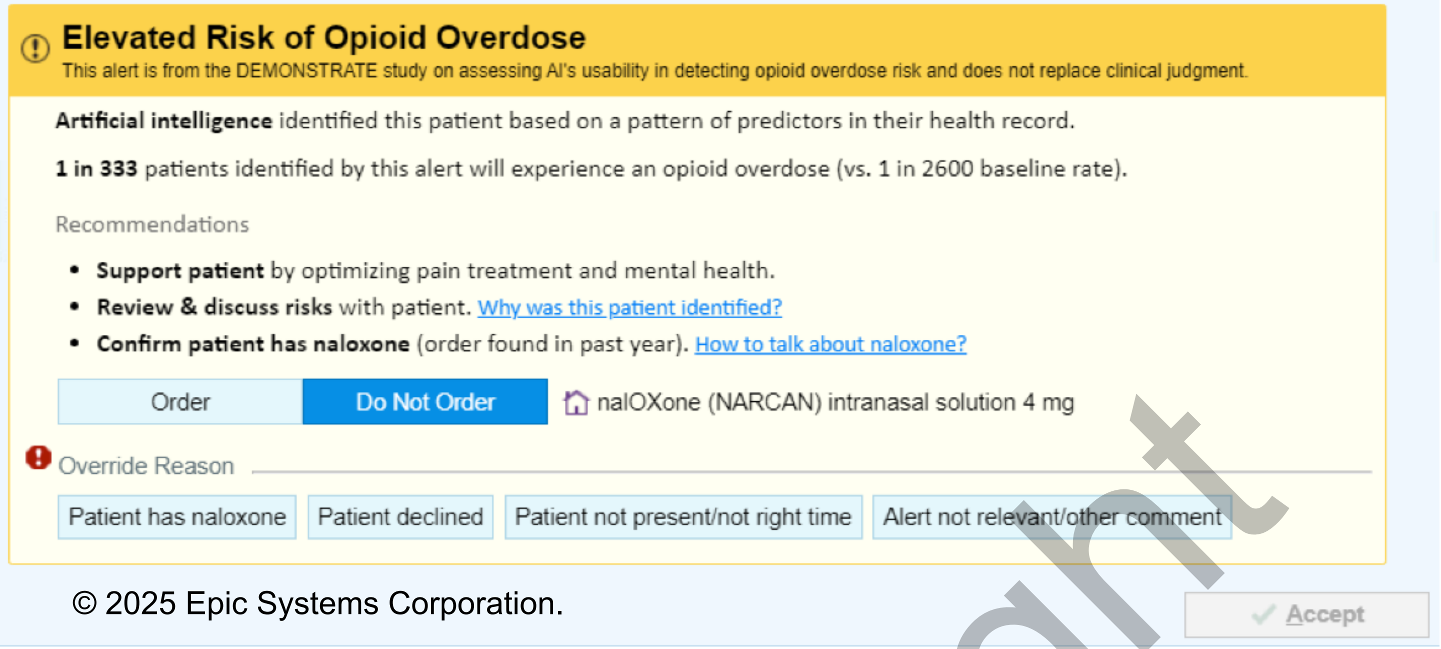

The 'Confirm Naloxone' alert will appear when <u>all</u> the following trigger criteria have been met.

- ✓ The algorithm determines patient has elevated risk of opioid overdose.
- ✓ An order for an opioid medication has been entered for the patient.
- ✓ Naloxone is <u>not</u> part of the current order.
- ✓ Naloxone <u>has</u> been ordered in the past year per Epic records.
- ✓ The provider signs the order (alert will appear on this action).

This alert is defaulted to "Do Not Order' naloxone. A provider must take an action to "Accept" the alert. An action can be ordering naloxone and/or selecting an override reason. Your selections and override reason determine the re-appearance criteria (see below) and are documented in the 'BPA Review' tab for one year.

Re-Appearance Criteria

If '**order**' is selected, the alert will appear again after 1 year (when the order typically expires).

If **'do not order'** is selected, the alert will appear again in 6 months. Selecting an override reason may change that timeframe:

- If you select '**patient has naloxone'**, the alert will appear again in 1 year.
- If you select '**patient declined'**, the alert will appear again in 6 months.
- If you select '**patient not present/not right time**', the alert will appear the next time the patient comes for a clinic visit.
- If you select '**alert not relevant/other reason**', the alert will appear again in 1 year.

More Details

Please see the Frequently Asked Questions and Patient Counseling Script on the following pages for more information.

© Copyright
