# Supplementary material for: Protocol for a Single-Arm Pilot Clinical Trial: Developing and Evaluating a Machine Learning Opioid Prediction & Risk-Stratification E-Platform (DEMONSTRATE)
Source: J Clin Med. 2025 Dec 1;14(23):8522. doi: 10.3390/jcm14238522 (PMC12693449; doi:10.3390/jcm14238522)
Supplement: Supplementary file 1 [file jcm-14-08522-s001.zip › Supplementary File S2_DEMONSTRATE FAQ Artificial Intelligence Alert Opioid Overdose Risk 20250904.pdf]

FAQs

ARTIFICIAL INTELLIGENCE ALERT OPIOID OVERDOSE RISK

Why was the patient identified?

An algorithm found a **pattern in electronic health record data** that suggests your patient has an **elevated risk of opioid overdose.**

This does **not** mean your patient is addicted to opioids, abusing opioids, or that they have previously overdosed. Increased risk can be caused by many things such as drug-drug interactions, and drug-condition interactions.

How do I interpret risk?

1 in 333

Patients identified by this alert will experience an opioid overdose in the next 3 months*

VS

1 in 2600

Baseline rate among UF Health patients with a previous opioid prescription*

*approximation

What data does the algorithm use?

The algorithm looks at patterns among **57 predictors** in the UF Health record, summarized below:

Patient Demographics

- Younger (e.g., <65)
- Male

Health Care Utilization

- More ED visits, outpatient visits, & hospitalizations.
- Received more procedures.
- Surgery for lost-time injury.

Prescriptions

- Prescription and/or overlap of:
  - Opioids
  - Muscle relaxants
  - Gabapentinoids
  - Benzodiazepines
- All non-opioid prescriptions combined.
- Buprenorphine for opioid use disorder.
- Antidepressant prescription.

Diagnoses

- Anemia
- Chronic pulmonary disease
- Coagulation deficiency
- Cognitive impairment
- Congestive heart failure
- Diabetes
- HIV/AIDs
- Hypertension
- Kidney or gall bladder stone
- Liver disease
- Mental health disorders
- Musculoskeletal disease
- Neurological disorders
- Non-prescription substance abuse
- Pain
- Paralysis
- Prescription drug abuse or misuse
- Renal failure
- Respiratory disease
- Total co-morbidity burden

© Copyright

How does the algorithm work?

1

First, the algorithm identified 57 predictors that are associated with patients who are admitted for opioid overdose at UF Health.

2

Next, the algorithm looks for these predictors among UF Health primary care clinic patients with a previous opioid prescription.

3

Patients are identified when the combined effect of their predictors suggests they are at elevated risk of opioid overdose. You see an alert when you order opioids for these patients.

What are the error rates of the algorithm?

OUT OF 25,000 PATIENTS

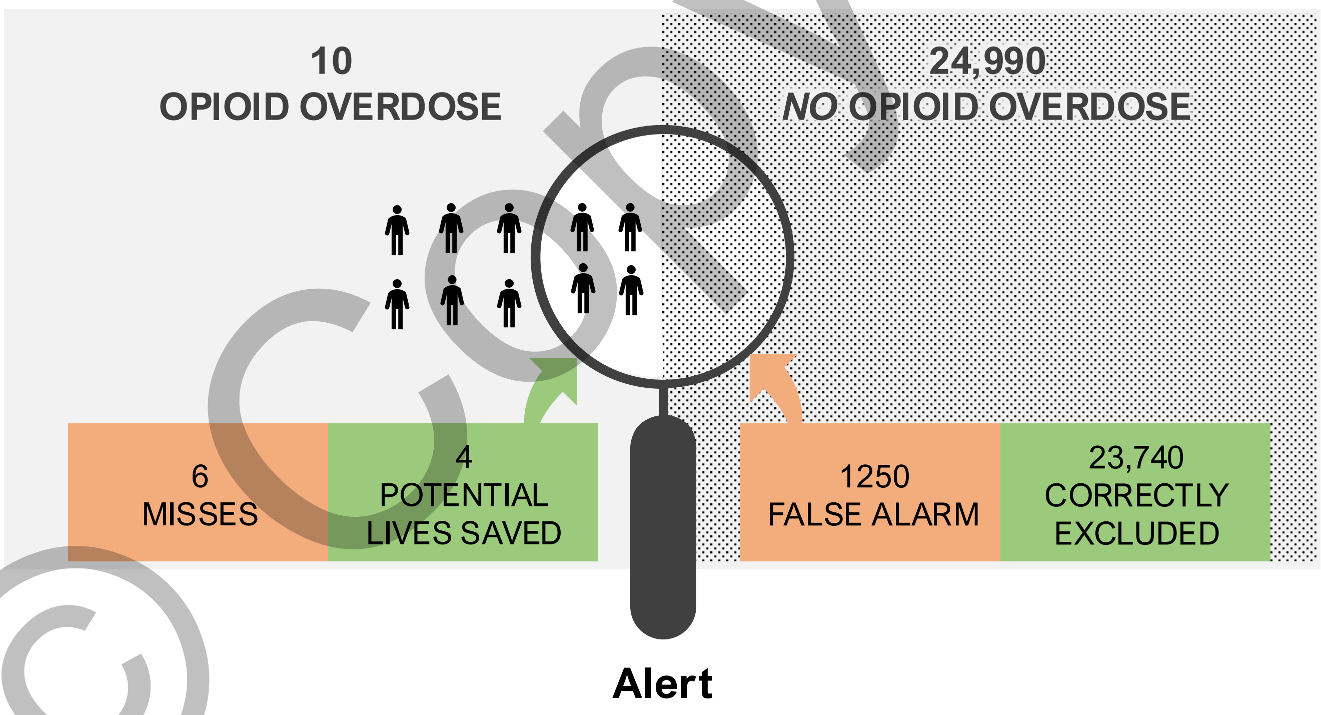

The error rates in the figure above are rounded to whole numbers, and not all patients with increased risk will have clinic visits. For that reason, you will see this alert less frequently than the numbers above suggest. If your patient panel is representative of the UF Health primary care clinic population, we estimate you will see this alert appear for about 1 in 50 patients with a previous opioid prescription. For every 333 patients identified by the alert, 1 is expected to experience an opioid overdose. Your response to the alert could prevent that overdose or prevent it from being fatal.

© Copyright

Is the algorithm validated?

Yes. The algorithm has been validated using multiple data sources such as Medicare and Medicaid claims data and electronic health records. The algorithm is updated and validated every six months based on its performance with real UF Health patients. The algorithm has been shown to **outperform current strategies** and measures that healthcare systems and payers are using to predict overdose risk.

Peer-reviewed Journal Articles:

- https://pubmed.ncbi.nlm.nih.gov/35623798/
- https://pubmed.ncbi.nlm.nih.gov/32678860/
- https://pubmed.ncbi.nlm.nih.gov/30901048/

Are my responses documented?

Yes. Your response to the alert is documented in the **'BPA Review' tab.**

This information is considered patient data and can be viewed by other providers and requested by the patient or patient's power of healthcare attorney. Additionally, your responses can be accessed by researchers and technology staff at UF Health. For example, they may download a report of responses to see how the alert is performing.

For more information, see the included tip sheet or email Debbie.Wilson@cop.ufl.edu.

Benefits vs. Harms

Benefits of identification include the opportunity for interventions that could reduce fatalities and emergency room visits.

VS

Harms of false alarms may include increased cost of naloxone prescribing, unnecessary changes to treatment, cost of more frequent follow-up, and potential stigma.

What should I do if I see this alert?

- Know that the algorithm cannot tease out individual predictors that put the patient at risk. It can only see the combined effect.
- Use your clinical expertise and relationship with the patient to evaluate the actual risk and make the best decision for your patient.
- The patient may benefit from extra support, close monitoring, treatment optimization, overdose prevention education, and naloxone prescribing.
- There are several effective forms of naloxone. The one in the alert (4mg nasal spray) is preferred by some because of the balance between cost, ease of use, and OTC availability.

Does the alert have limitations?

Faulty or missing data from EHR: The algorithm looks at data in the UF Health electronic health record only. If the underlying data is wrong or missing, the algorithm won't know that.

Excluded patients: Only patients with a previous opioid prescription at UF Health are included in the algorithm. Patients with malignant cancer or in end-of-life care are excluded from the algorithm. The alert will not appear for these patients, even if their underlying risk is elevated.

Bias: Assessments found no significant race or gender bias with the algorithm.

© Copyright
