# Supplementary material for: Protocol for a Single-Arm Pilot Clinical Trial: Developing and Evaluating a Machine Learning Opioid Prediction & Risk-Stratification E-Platform (DEMONSTRATE)
Source: J Clin Med. 2025 Dec 1;14(23):8522. doi: 10.3390/jcm14238522 (PMC12693449; doi:10.3390/jcm14238522)
Supplement: Supplementary file 1 [file jcm-14-08522-s001.zip › Supplementary File S3_DEMONSTRATE 30 Second Patient Counseling Scripts 20250904.pdf]

30-SECOND PATIENT COUNSELING SCRIPTS

Opioid Risks

One risk with opioid medication is it can cause breathing to slow or stop.

This can happen, even at prescribed doses.

Or accidentally, if you take too much or take with things that make you sleepy or relaxed.

Another risk is that a family member or child could accidentally swallow it.

Naloxone

As a precaution, we offer naloxone. It's a medication that can reverse the effect of opioids and is safe to give to anyone.

Having it makes sure you are prepared for the worst. Just like a fire extinguisher or first aid kit, make sure those around you know where it is and how to use it.

Do you have any questions or concerns about naloxone or getting it from the pharmacy?

Additional Information

Opioids can provide meaningful pain relief but also have serious risks such as addiction and overdose. Even at prescribed doses, opioid medications carry the risk of addiction, overdose, and death.

1. Because of these serious risks, it's important to be very aware of how to take your opioid medication safely.
2. Take your opioid medicine exactly as prescribed. Do not cut, break, chew, crush, or dissolve your medicine.
3. Call your healthcare provider if the opioid medication is not controlling your pain. Do not increase the dose on your own.
4. Call your healthcare provider if the opioid medication causes side effects such as constipation, nausea, dizziness, drowsiness, or breathing difficulty.
5. Avoid taking alcohol or certain medications with your opioid medication as these may cause you to stop breathing, which can lead to death. These medications include:
   a) Benzodiazepines like diazepam (VALIUM), alprazolam (XANAX), or lorazepam (ATIVAN)
   b) Gabapentinoids like gabapentin (NEUROTIN) or pregabalin (LYRICA)
   c) Muscle relaxants like cyclobenzaprine (FLEXERIL) or methocarbamol (ROBAXIN)
   d) Sleep medicines like zolpidem (AMBIEN) or eszopiclone (LUNESTA)
   e) Other prescription opioid medicines
6. Have naloxone on hand and make sure those around you know where it is and how to use it.

© Copyright

© Copyright
