# Supplementary material for: Protocol for a Single-Arm Pilot Clinical Trial: Developing and Evaluating a Machine Learning Opioid Prediction & Risk-Stratification E-Platform (DEMONSTRATE)
Source: J Clin Med. 2025 Dec 1;14(23):8522. doi: 10.3390/jcm14238522 (PMC12693449; doi:10.3390/jcm14238522)
Supplement: Supplementary file 1 [file jcm-14-08522-s001.zip › Supplementary File S4_DEMONSTRATE Opioid Overdose Risk Pocket Card 20250904.pdf]

COMING SOON

You’ll be the first to see

UF Health’s first AI driven alert

Elevated Risk of Opioid Overdose

This alert is from the DEMONSTRATE study on assessing AI's usability in detecting opioid overdose risk and does not replace clinical judgment.

Artificial intelligence identified this patient based on a pattern of predictors in their health record.

1 in 333 patients identified by this alert will experience an opioid overdose (vs. 1 in 2600 baseline rate).

Recommendations

- **Support patient** by optimizing pain treatment and mental health.
- **Review & discuss risks** with patient. Why was this patient identified?
- **Offer naloxone** yearly (order not found in past year). How to talk about naloxone?

Order | Do Not Order | **nalOXone (NARCAN) intranasal solution 4 mg**

Or select an override reason

Patient has naloxone | Patient declined | Patient not present/not right time | Alert not relevant/other comment

© 2025 Epic Systems Corporation.

Accept

© Copyright

Quick Facts

- **When will it appear?** When you sign orders for opioids, <u>only if</u> the artificial intelligence algorithm identifies the patient as having elevated risk of opioid overdose.
- **What is the benefit?** It is expected to appear less frequently than the legacy naloxone alert and be more precise at identifying patients at elevated risk of opioid overdose. Your response to the alert could save a life!
- **What is the risk?** The alert could be a false alarm. The alert does <u>not</u> mean the patient is addicted or has previously overdosed. The algorithm uses over 50 predictors in the health record to assess risk, including demographics, health care utilization, prescriptions, and diagnoses.
- **How do I respond?** To order naloxone, click ‘Accept’. To dismiss the alert, select ‘Do Not Order’, then select an ‘Override Reason’, and click ‘Accept’
- **Who do I contact with questions?** Debbie.Wilson@cop.ufl.edu

© Copyright

© Copyright
