# Supplementary material for: Protocol for a Single-Arm Pilot Clinical Trial: Developing and Evaluating a Machine Learning Opioid Prediction & Risk-Stratification E-Platform (DEMONSTRATE)
Source: J Clin Med. 2025 Dec 1;14(23):8522. doi: 10.3390/jcm14238522 (PMC12693449; doi:10.3390/jcm14238522)
Supplement: Supplementary file 1 [file jcm-14-08522-s001.zip › Supplementary File S5_DEMONSTRATE CDC Guidelines on Opioid Prescribing Infographic 20250904.pdf]

Prescribing Opioids for Pain

Adapted from the CDC 2022 Clinical Practice Guideline for Prescribing Opioids for Pain: Guideline Recommendations and Guiding Principles

Evaluating opioid use for pain management

- Prioritize non-opioid and nonpharmacologic treatment.
- Consider opioids only if expected benefits outweigh risks.
- Before starting opioids:
    - Discuss realistic benefits and known risks with patients.
    - Set treatment goals for pain and function.
    - Plan for potential discontinuation if risks outweigh benefits.

Opioid Selection & Dosage

- Start with immediate-release opioids
- Initiate with the lowest effective dose, especially for opioid-naïve patients
- If continued, regularly assess benefits vs. risks before increasing dosage.
- Avoid abrupt discontinuation or rapid dose reductions unless life-threatening risks (e.g., overdose warning signs) are present.
- If opioids are continued:
    - Optimize non-opioid therapies alongside opioid treatment.
    - If risks outweigh benefits, gradually taper or discontinue opioid safely.

Duration & Follow-up

- Prescribe only as much as needed for acute pain.
- Re-assess benefits and risks within 1–4 weeks of starting or escalating therapy
- Regularly re-evaluate benefits and risks of continued therapy.

© Copyright

Risk Assessment & Harm Reduction

- Evaluate opioid-related risks before and periodically during therapy.
- Include risk mitigation strategies such as offering naloxone in the management plan.
- Use state prescription drug monitoring program (PDMP) data to review the current use of controlled substance prescriptions and prior history.
- Consider toxicology testing to monitor for prescribed and nonprescribed controlled substances.
- Use caution when prescribing opioids with benzodiazepines or other central nervous system depressants.
- Offer or arrange evidence-based medications for opioid use disorder (MOUD) for patients with OUD:
  - Buprenorphine, methadone, and naltrexone are FDA-approved for OUD.
  - Buprenorphine can be prescribed by any clinician with a current, standard DEA registration with Schedule III authority, in any clinical setting.
  - Naltrexone can be prescribed by any licensed provider.
- Detoxification alone, without MOUD is not recommended due to high risk of relapse and overdose.

Guiding Principles

1. Pain should be appropriately assessed and managed, whether opioids are used or not.
2. Guidelines are voluntary and should support, not replace, individualized, patient-centered care.
3. A multimodal and multidisciplinary approach is crucial for effective pain management.
4. Avoid misapplying guidelines or implementing rigid policies that could cause unintended harms or consequences.
5. Ensure equitable access to an effective, affordable, coordinated and culturally appropriate pain management (nonpharmacologic and pharmacologic).

©University of Pittsburgh ©Applied Decision Science LLC.
Adapted from: Dowell D, Ragan KR, Jones CM, Baldwin GT, Chou R. CDC Clinical Practice Guideline for Prescribing Opioids for Pain - United States, 2022. MMWR Recomm Rep. 2022 Nov 4;71(3):1-95. doi: 10.15585/mmwr.rr7103a1. PMID: 36327391; PMCID: PMC9639433. https://www.cdc.gov/mmwr/volumes/71/rr/rr7103a1.htm

© Copyright

©Copyright
