# Supplementary material for: Protocol for a Single-Arm Pilot Clinical Trial: Developing and Evaluating a Machine Learning Opioid Prediction & Risk-Stratification E-Platform (DEMONSTRATE)
Source: J Clin Med. 2025 Dec 1;14(23):8522. doi: 10.3390/jcm14238522 (PMC12693449; doi:10.3390/jcm14238522)
Supplement: Supplementary file 1 [file jcm-14-08522-s001.zip › Supplementary File S6_DEMONSTRATE_Post-Implementation Questionnaire 20250904.pdf]

File S6. Post-Implementation Questionnaire

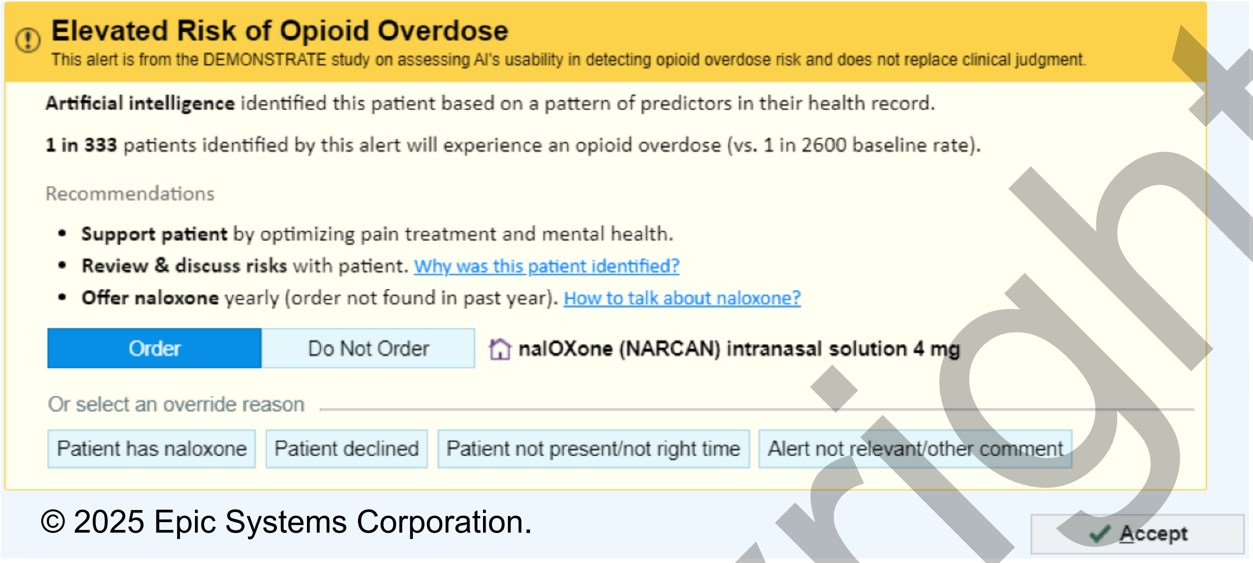

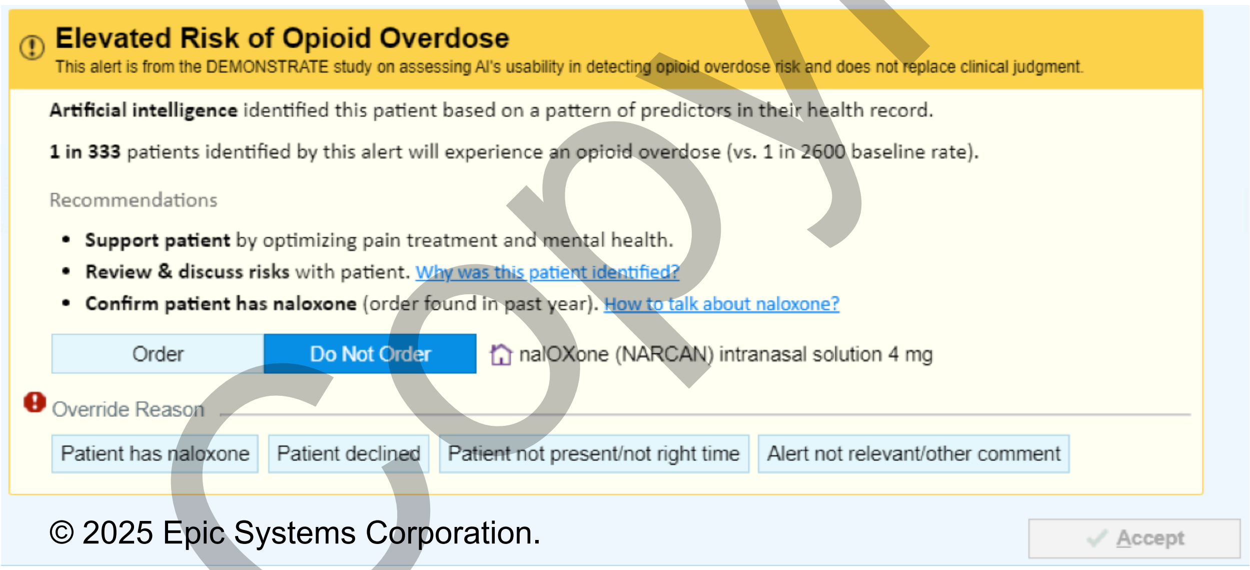

1. Have you seen one of the alerts above? [Yes or No. If no, they don't need to complete survey]
2. How many times have you seen this alert? [Once, More than once]

© Copyright

For the following questions, please indicate how much you agree with the statement. Please answer based on your most recent experience with the alert.
[scale = strongly disagree, disagree, agree, strongly agree]

3. The information provided by the alert was clear. [usability]
4. The alert was easy to use. [usability]
5. The alert helped me identify a patient at increased risk of opioid overdose. [right person]
6. The alert helped me understand the patient's opioid overdose risk. [right info]
7. The alert provided recommendations that helped me manage the opioid overdose risk of the identified patient. [right info]
8. The alert identified the right patient with elevated overdose risk. [right person]
9. I'm the right health care team member to be notified that the patient has elevated risk of opioid overdose. [right person]
10. A "pop-up" alert is the right approach to notify me that the patient has elevated risk of opioid overdose. [right format/channel]
11. The right time to notify me that my patient has elevated risk of opioid overdose is when I sign an opioid order. [right time]
12. The frequency of the alert's appearance is just right [right time]
13. [If answered disagree or strongly disagree] I would rather the alert appear [less frequently or more frequently]
14. I prefer this alert to the other naloxone alert (pictured below)?

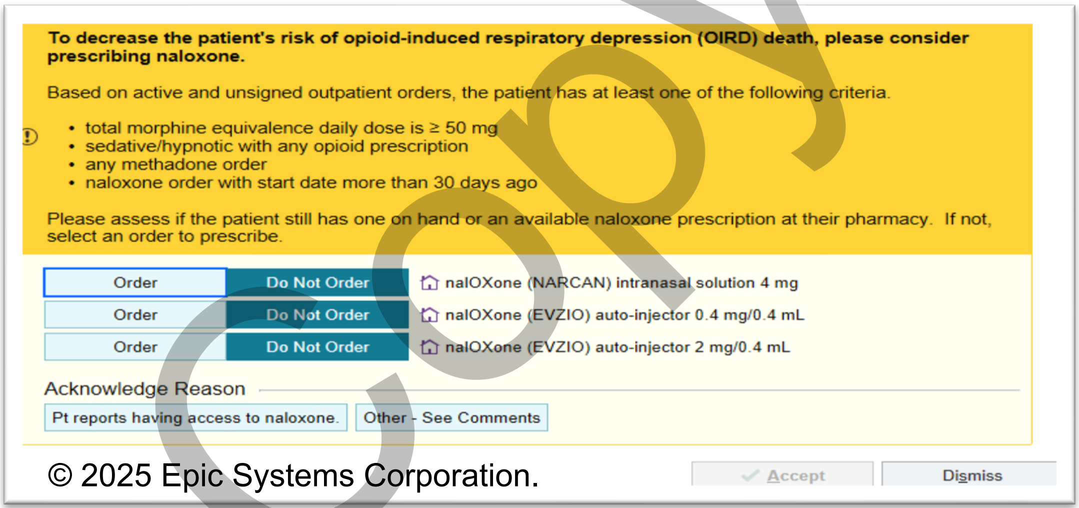

15. I would like this alert to continue to operate in Epic.
16. Is there anything else you would like to record about your experience with the alert before our interview? [Open ended]

© Copyright
