# Supplementary material for: Protocol for a Single-Arm Pilot Clinical Trial: Developing and Evaluating a Machine Learning Opioid Prediction & Risk-Stratification E-Platform (DEMONSTRATE)
Source: J Clin Med. 2025 Dec 1;14(23):8522. doi: 10.3390/jcm14238522 (PMC12693449; doi:10.3390/jcm14238522)
Supplement: Supplementary file 1 [file jcm-14-08522-s001.zip › Supplementary File S7_DEMONSTRATE_Post-Implementation Interview Guide 20250904.pdf]

File S7. Post-Implementation Interview Guide

Interview Questions:

1. Was the alert relevant to this patient case?
   a. What made it relevant/irrelevant?
2. What was your response to the alert?
   a. Did you talk to the patient, review the record, change treatment plan, talk to other members of the health care team?
3. Does knowing this alert was generated by AI influence your perception of how well it will identify patients who are truly at elevated risk?
4. Does this alert change the way you view the patient? In what way?
5. What is most useful about this alert?
6. What would make the alert more useful?

[Any survey questions that received a negative response should be probed in interview. "You indicated that BPA alert is not the right approach to notify you. Tell me more about that."]

© Copyright

© Copyright
