# Supplementary material for: Protocol for a Single-Arm Pilot Clinical Trial: Developing and Evaluating a Machine Learning Opioid Prediction & Risk-Stratification E-Platform (DEMONSTRATE)
Source: J Clin Med. 2025 Dec 1;14(23):8522. doi: 10.3390/jcm14238522 (PMC12693449; doi:10.3390/jcm14238522)
Supplement: Supplementary file 1 [file jcm-14-08522-s001.zip › Supplementary File S8_DEMONSTRATE Alert outcome measures_manuscript version.pdf]

File S8. Alert Use-Related Outcome Measures

Alert use-related measures*	Numerator	Denominator	Timeframe post-implementation	Data Sources	Implementation Concept
Total no. alert appearances (How many times did an alert appear?)	N/A	N/A	3, 6, 12 months	EPIC Workbench Report	Penetration
Total no. 'Offer Naloxone' alerts	N/A	N/A	3, 6, 12 months	EPIC Workbench Report	Penetration
Total no. 'Confirm Naloxone' alerts	N/A	N/A	3, 6, 12 months	EPIC Workbench Report	Penetration
% of patients received an alert of patients flagged as elevated risk	Unique patients with ≥1 alert	ML-flagged elevated-risk patients	Weekly tracking & 3, 6, 12 months	EPIC Workbench Report + EHR + risk score data	Penetration
No. average alerts per alerted patient	Total alerts	Unique alerted patients	Weekly tracking & 3, 6, 12 months	EPIC Workbench Report	Penetration
% of PCPs who received ≥1 alert	Unique PCPs receiving alerts	Total PCPs in clinics	Weekly tracking & 3, 6, 12 months	EPIC Workbench Report	Penetration
% of alert resulted in accepted naloxone order (Did the alert result in a click to accept the naloxone order?	No. times of alerts where order accepted	Total alerts appeared	3, 6, 12 months	EPIC Workbench Report	Adoption
For each patient that got an alert, % patient-level naloxone acceptance (i.e., did the alert result in a click to accept the naloxone order?)	Patients with ≥1 accepted naloxone order	Patients receiving alerts	3, 6, 12 months	EPIC Workbench Report	Adoption
% alerts with accepted order but unsigned naloxone orders (i.e., Did PCP delete naloxone order after clicking accept?)	No. alerts that had naloxone order selected but unsigned naloxone order.	Number of alerts that had naloxone order selected.	3, 6, 12 months	EPIC Workbench Report + Chart review	Adoption
% of alerts with an override reason submitted "Patient has naloxone	No. alerts with "Patient has naloxone" override	No. alerts where override was selected	3, 6, 12 months	EPIC Workbench Report + Chart review	Appropriateness
% of alerts with an override reason submitted "Patient declined"	No. alerts with "Patient declined" override	No. alerts where override was selected	3, 6, 12 months	EPIC Workbench Report + Chart review	Appropriateness
% of alerts with an override reason submitted "Patient not present/not the right time"	No. alerts with "Patient not present/not the right time" override	No. alerts where override was selected	3, 6, 12 months	EPIC Workbench Report + Chart review	Appropriateness
% of alerts with an override reason submitted ("Alert not relevant/other)	No. alerts with "alert not relevant/other comment" override	No. alerts where override was selected	3, 6, 12 months	EPIC Workbench Report + Chart review	Appropriateness
% of alerts with an override comment submitted (with qualitative analyses)	No. alerts with text comments submitted	Alerts with 'other' override	12 months	EPIC Workbench Report + Chart review	Appropriateness
Thumbs-up counts	Thumbs-up	Total alerts	12 months	EPIC Workbench Report	Appropriateness

Abbreviations: EHR: electronic health records; ML: machine-learning; No.: number of PCP: primary care providers;

***: We will conduct similar alert use-related measure analysis for the existing legacy alert.**
